# Spherical Video‐Based Virtual Reality for Nurses' Workplace Violence Management: A Convergent Mixed‐Methods Study

**DOI:** 10.1111/jan.70563

**Published:** 2026-02-24

**Authors:** Yen‐Yin Lin, Ting‐Ti Lin, Yin‐Ling Hung, Hsin‐Pei Feng, Chun‐Yu Liang, Wen‐Chii Tzeng

**Affiliations:** ^1^ Tri‐Service General Hospital Taipei City Taiwan; ^2^ National Defense Medical University Taipei City Taiwan

**Keywords:** immersive learning, mixed‐methods research, nurse, nursing education, virtual reality, workplace violence

## Abstract

**Aim:**

To evaluate the feasibility, effectiveness, and acceptability of a spherical video‐based virtual reality training programme aimed at helping nurses manage workplace violence.

**Design:**

A convergent mixed‐methods study.

**Methods:**

This study included nurses from a tertiary medical centre in Taiwan. The training programme involved four interactive 360° scenarios focused on recognising, de‐escalating, and responding to workplace violence. Quantitative measures included risk perception, confidence in coping with aggression, and technology acceptance. Qualitative measures included the participants' learning experiences. Quantitative and qualitative findings were integrated through joint displays.

**Results:**

The programme was feasible, with all participants completing the training. Nurses reported high levels of perceived usefulness and ease of use. Quantitative data revealed considerable improvements in risk awareness and confidence in responding to incidents of violence. Qualitative data revealed that immersion and emotional resonance enhanced engagement, fostered self‐reflection, and reinforced learning. Technical challenges included subtitle placement and speech recognition accuracy.

**Conclusion:**

Spherical video‐based virtual reality is a feasible, acceptable, and effective training approach that improves nurses' preparedness for managing workplace violence by enhancing situational awareness and confidence in addressing high‐risk situations.

**Implications for the Profession and/or Patient Care:**

Integrating spherical video‐based virtual reality into continual education may strengthen nurses' workplace safety competencies, prevent harm from incidents of violence, and improve patient care in stressful environments.

**Impact:**

Workplace violence undermines nurse safety and patient care. Current training modules often lack contextual realism. Our programme improved nurses' awareness, confidence, and reflective learning and was feasible and well accepted. The findings are relevant to nursing educators, hospital administrators, and policymakers seeking sustainable strategies for addressing workplace violence.

**Reporting Method:**

This study adhered to the Revised Standards for Quality Improvement Reporting Excellence.

**Patient or Public Contribution:**

Patients or the public were not involved in the design, conduct, or reporting of this study.

## What Does This Paper Contribute to the Wider Global Clinical Community?

- Our study highlights spherical video‐based virtual reality as an innovative modality for helping nurses effectively manage workplace violence.
- It demonstrates how immersive technologies can increase situational awareness and support reflective practice.
- It further offers practical directions for implementing scalable, technology‐enhanced interventions in diverse healthcare contexts.

## 1 Introduction

Workplace violence is a major global public health concern that undermines the physical safety, psychological well‐being, and professional performance of healthcare workers (Al‐Qadi 2020; Wang et al. 2022). The World Health Organization (2022) estimated that approximately 62% of all healthcare professionals worldwide have experienced some form of workplace violence. Because of their continual and close interactions with patients and patient families, nurses are disproportionately vulnerable to workplace violence. A large‐scale cross‐sectional study of 2626 nurses in Taiwan reported that 70.6% of the cohort had experienced physical or psychological violence in the preceding year (Niu et al. 2018). Beyond causing immediate harm, workplace violence reduces job satisfaction, employee retention, patient care, and safety (Stafford et al. 2022), establishing itself as a pressing nursing and health policy concern.

## 2 Background

The causes of workplace violence are multifactorial, spanning individual, organisational, and systemic domains—for example, occupational stress, institutional culture, and environmental constraints (Sheppard et al. 2022). Workplace violence is often underreported because of limited legal literacy, cumbersome reporting procedures, and inadequate organisational support (Dafny et al. 2025). These barriers hinder timely implementation of preventive and disciplinary measures, highlighting the need for sustained educational strategies to improve nurses' ability to effectively recognise, de‐escalate, and respond to violence.

Traditional workplace violence training programmes typically include lectures, electronic learning modules, role‐play sessions, video demonstrations, or simulation‐based activities to teach risk assessment, verbal de‐escalation, and self‐protection skills (Lamont and Brunero 2018; Story et al. 2020). Some scholars have developed multicomponent curricula covering skills essential for identifying high‐risk scenarios, engaging in effective communication, and managing conflicts (Chang et al. 2022; Ming et al. 2019). Courses involving high‐fidelity simulations aimed at enhancing nurses' decision‐making and situational responses under pressure have also been developed (Young et al. 2022). Although learner feedback has generally been positive for currently available programmes, a systematic review revealed inconsistent outcomes and limited transferability to real‐world practice because of insufficient contextual realism and resource‐intensive delivery (Dafny et al. 2025). These limitations reduce the feasibility of implementing such programmes as sustainable, routine interventions.

Workplace violence is a highly context‐dependent phenomenon, and the risks faced by nurses vary across clinical settings. Hence, training should prioritise improving nurses' judgement in dynamic situations. Christensen et al. (2024) concluded that nurses' responses to violent encounters are shaped by their knowledge regarding violence management and their immediate emotional reactions. Effective responses require increased sensitivity to environmental cues and interpersonal dynamics—competencies that cannot be cultivated through classroom instruction or text‐based materials alone. This gap necessitates innovative, sustainable, and immersive learning approaches.

Spherical video‐based virtual reality (SVVR) is an interactive learning technique that uses 360° immersive videos and a first‐person perspective to create a heightened sense of presence and emotional engagement for learners (Baysan et al. 2023). In this approach, learners use a head‐mounted display and immerse themselves in dynamic, interactive environments that support real‐time observation, situational judgement, and reflective thinking. This modality is particularly suitable for simulating high‐stress, low‐frequency, and high‐risk clinical scenarios. SVVR has several pedagogical advantages, including repeatability, an environment conducive to trial and error, and active learner participation. Lee et al. (2024) developed an SVVR module focused on caring for patients with schizophrenia that was designed to train nursing students in recognising and managing psychiatric symptoms, suicide risk, and violent behaviour; the module effectively increased learner engagement and reduced fear and stigma towards individuals with mental illness. Similarly, Huang et al. (2022) incorporated SVVR into a transfusion training programme for novice nurses; this programme considerably improved nurses' clinical judgement and self‐efficacy. The feasibility of this programme for workplace violence training remains to be systematically investigated. Evidence regarding the feasibility, effectiveness, and acceptability of SVVR interventions aimed at managing workplace violence remains limited, warranting empirical studies.

Investigating outcomes of novel, technology‐enhanced training programmes requires capturing both measurable outcomes and nuanced learner experiences. A convergent mixed‐methods design is well suited for this purpose, given that it enables the integration of quantitative data on feasibility, risk perception, and confidence with qualitative data on acceptance, engagement, and contextual challenges. This design provides complementary evidence for investigating not only whether SVVR‐assisted training is effective but also how and why it is perceived as useful or feasible in clinical practice (Creswell and Plano Clark 2018; Younas and Durante 2023).

## 3 The Study

In this convergent mixed‐methods study, we evaluated the feasibility, effectiveness, and acceptability of an SVVR workplace violence management training programme for nurses. The specific objectives were as follows: (1) to determine the feasibility of implementing the aforementioned programme in clinical settings, (2) to assess postintervention changes in nurses' risk perceptions and confidence in responding to workplace violence; and (3) to explore nurses' intervention acceptance and learning experiences.

Based on these objectives, the study was guided by the following research questions (RQs):

Is the SVVR‐based workplace violence management training programme feasible to implement in clinical nursing settings?

Does participation in the SVVR training programme result in changes in nurses' perceived risk and confidence in responding to workplace violence?

How do nurses perceive the acceptability, learning experiences, and practical relevance of the SVVR training programme?

## 4 Methods

### 4.1 Study Design

This study employed a convergent mixed‐methods design (Creswell and Plano Clark 2018) and was reported in accordance with the Revised Standards for Quality Improvement Reporting Excellence (SQUIRE 2.0), as it evaluated the feasibility, effectiveness, and acceptability of an educational intervention in a clinical setting. Quantitative and qualitative data were collected concurrently, analysed separately, and then integrated through merging and comparison. The results are presented in joint displays to facilitate meta‐inferences reflecting both measurable outcomes and experiential insights. This study was conducted between February and March 2025.

### 4.2 Study Setting and Sampling

Eligible participants were recruited using purposive sampling from a medical centre in northern Taiwan that has a high risk of workplace violence (because it offers acute and high‐intensity clinical services).

The required minimum sample size was calculated using G*Power software (version 3.1.9.7). Power analysis was performed under the assumption of a paired‐sample *t*‐test; a moderate effect size (Cohen's *d* = 0.50), as reported by Huang et al. (2022) and based on a similar training intervention, was used. With a significance level of *α* = 0.05 and statistical power (1–*β*) = 0.80, it was calculated that at least 34 participants were required for the analysis.

To account for potential attrition, 62 nurses were approached. After telephonic screening and research‐related briefing, one individual was excluded because of not meeting the eligibility criteria. Seven individuals declined participation because of scheduling conflicts or procedural concerns. In total, 54 nurses completed the training programme. This number provides sufficient power for the planned analyses under the predetermined assumptions.

### 4.3 Eligibility Criteria

The inclusion criteria were as follows: being aged 20–65 years, being licensed and currently practising as a registered nurse, and holding frontline positions that necessitate direct patient care. The exclusion criteria were as follows: serving in managerial roles or roles not involving direct patient care; having a history of epilepsy, cardiovascular disease, or vertigo; receiving a diagnosis of anxiety disorder, panic disorder, or claustrophobia; and having myopia exceeding −6.00 dioptres or a history of ocular surgery.

### 4.4 Study Intervention

The SVVR intervention was developed using the ADDIE instructional design model, which comprises five phases: analysis, design, development, implementation, and evaluation (Molenda 2015). In the initial phase, the research team, which included two experienced clinical nurses and two researchers with expertise in virtual‐reality‐based curriculum design, reviewed the literature (Chang et al. 2022; Ming et al. 2019; Story et al. 2020) and assessed learner needs to define learning objectives and core competencies. To ensure contextual relevance and authenticity, the programme content was derived from real cases of workplace violence.

**FIGURE 1:**
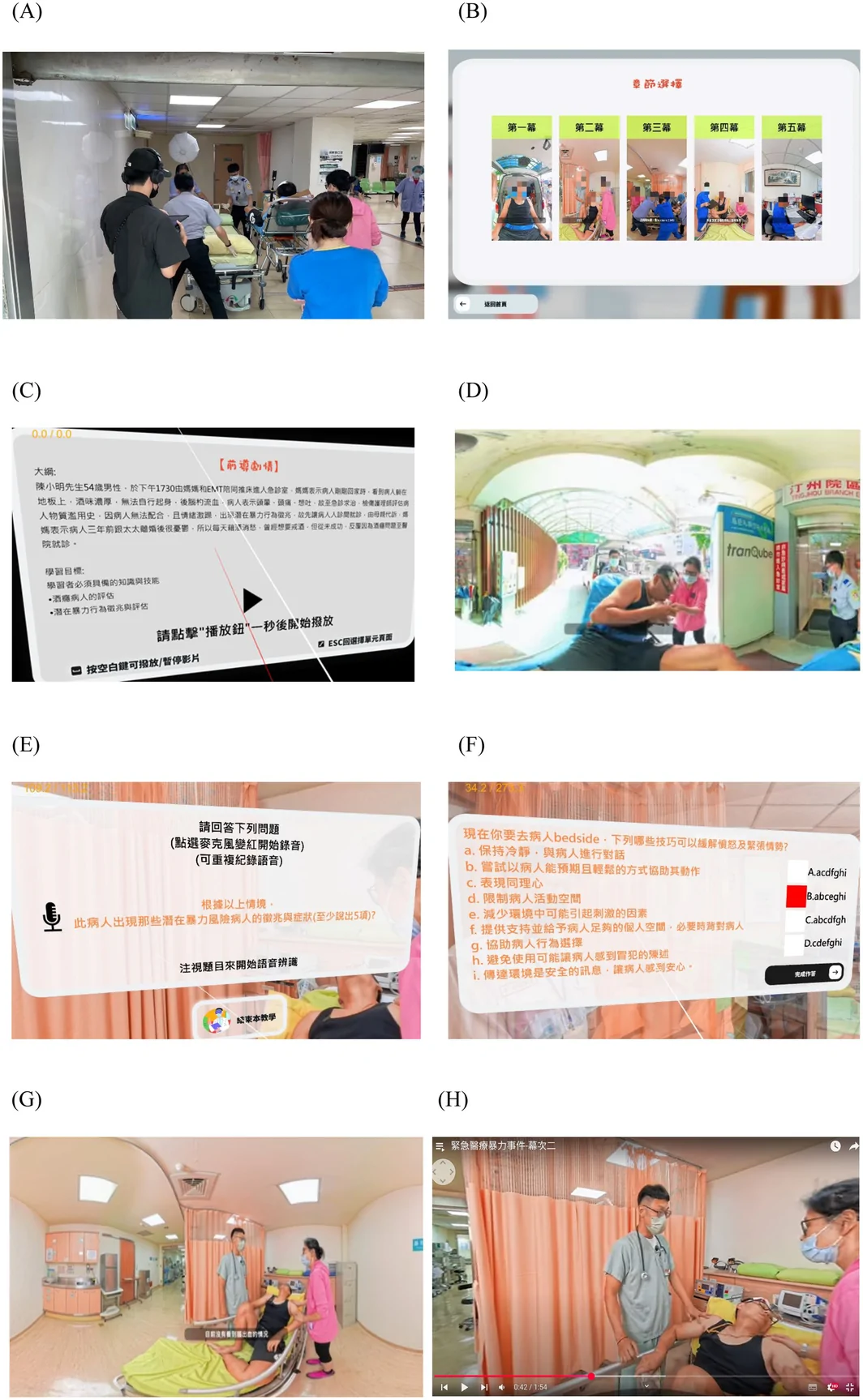
Development of and screenshots from spherical video‐based virtual reality training programme for managing workplace violence. (A) Behind‐the‐scenes view of scenario filming with Insta360 X4 panoramic camera. (B) Module selection interface on web‐based learning platform. (C) Scenario introduction scene presenting the incident context and learning objectives. (D) Immersive simulation scene depicting patient with alcohol intoxication being transported to emergency department via ambulance; the patient was accompanied by his sister. (E) Screenshot of interactive voice‐response interface prompting learners to provide verbal responses. (F) Screenshot of interactive multiple‐choice question interface; participants responded using handheld controllers. (G) Screenshot from immersive spherical video‐based virtual reality programme. (H) Comparison screenshot from standard two‐dimensional video, highlighting differences in learner perspective and immersion.

The final product was a 20‐min immersive 360° video simulating the management of an alcohol‐intoxicated patient in an emergency department (Figure 1). The scenario comprised five segments: initial assessment, violence risk recognition, verbal de‐escalation, response to a violent incident, and postincident management. Each segment incorporated voice‐interactive prompts requiring the participants to make situational decisions by using natural language—for example, ‘Identify five potential indicators of violence’, ‘What preventive measures would you take in the face of escalating risk?’, and ‘How would you calm an agitated patient?’

The instructional video was collaboratively developed by the research team and a digital technology firm by using an Insta360 X4 panoramic camera and deployed on the internal web‐based learning platform of the principal investigator's institute. The intervention was delivered using an HTC VIVE XR Elite head‐mounted display with two motion‐tracking controllers, spatial positioning, and three‐dimensional audio. The six‐degrees‐of‐freedom controllers, which were equipped with accelerometers and gyroscopes, enabled real‐time motion tracking, allowing users to navigate the interactive scenario and respond using voice commands. Voice inputs were captured and analysed using integrated speech recognition technology. The first author monitored each session in real time and conducted a structured debriefing immediately after the intervention. The total intervention time was approximately 60 min per participant.

### 4.5 Intervention Fidelity

Fidelity of the SVVR intervention was maintained through multiple strategies. First, the programme content was validated by an expert panel comprising three nursing scholars and one emergency physician. The overall content validity index was 0.92, indicating high content relevance and appropriateness of learning objectives. Second, to minimise variability in implementation, all sessions were delivered in a standardised manner by using the same hardware, software, and instructional protocol. Third, the first author, with clinical expertise in psychiatric and emergency nursing, monitored each session to ensure procedural adherence and address potential technical or procedural challenges. Finally, individual debriefing was conducted immediately after each session to reinforce learning objectives, clarify correct violence management procedures, and ensure consistency in the participants' learning experiences.

### 4.6 Measures and Instruments

#### 4.6.1 Demographic Characteristics

Demographic variables included age, sex, marital status, educational attainment, work unit, clinical ladder level, work duration (years of nursing experience), prior exposure to workplace violence, and workplace violence management training experience.

#### 4.6.2 Completion Metrics

Completion metrics included programme completion rate, measurement completion rate, and attrition rate.

#### 4.6.3 Perception of Aggression Scale

The Perception of Aggression Scale (POAS), originally developed by Jansen, was translated and adapted into an 18‐item Chinese version by Ming (2017). Responses to each item on the POAS are rated on a 5‐point Likert scale, with endpoints ranging from 1 (*strongly disagree*) to 5 (*strongly agree*). The total score ranges from 18 to 90. Higher scores indicate greater awareness and a more comprehensive understanding of workplace violence. In the present study, Cronbach's α for the POAS was 0.83.

#### 4.6.4 Confidence in Coping With Patient Aggression Scale

The Confidence in Coping with Patient Aggression (CCPA) scale was used to evaluate nurses' confidence in coping with patients' aggressive behaviour. The CCPA scale, originally developed by Thackrey, was translated and adapted into a 17‐item Chinese version by Ming (2017). Responses to each item of the CCPA scale are rated on a 5‐point Likert scale, with endpoints ranging from 1 (*strongly disagree*) to 5 (*strongly agree*). The total score ranges from 17 to 85. Higher scores indicate greater confidence in coping with violent incidents. In the present study, Cronbach's α for the CCPA scale was 0.91.

#### 4.6.5 Virtual Reality Sickness Questionnaire

The Virtual Reality Sickness Questionnaire (VRSQ), adapted by Kim et al. (2018) from the Simulator Sickness Questionnaire, comprises an oculomotor subscale (four items) and a disorientation subscale (five items). Responses to each item on the VRSQ are rated on a 4‐point Likert scale, with endpoints ranging from 0 (*none*) to 3 (*severe*). The total score is calculated using the following formula: [(oculomotor subscale score/12) × 100 + (disorientation subscale score/15) × 100]/2. Higher VRSQ scores indicate greater discomfort. In the present study, Cronbach's α values for the oculomotor and disorientation subscales were 0.72 and 0.67, respectively.

#### 4.6.6 Technology Acceptance Questionnaire

The Technology Acceptance Questionnaire (TAQ), developed by Hwang et al. (2013), has two subscales: perceived usefulness (six items) and perceived ease of use (seven items). Responses to each item on the TAQ are rated on a 5‐point Likert scale, with endpoints ranging from 1 (*strongly disagree*) to 5 (*strongly agree*). The total score ranges from 5 to 65. In the present study, Cronbach's α values for the perceived usefulness and perceived ease of use subscales were 0.93 and 0.96, respectively.

#### 4.6.7 Semistructured Interviews

Semistructured interviews, each lasting 15–20 min, were conducted in a quiet, private setting. Interview questions covered four domains: (1) perceived differences and similarities between the SVVR intervention and previous training programmes, (2) major learning gains, (3) suggestions for programme improvement, and (4) willingness to recommend the programme to colleagues and reasons for doing so.

### 4.7 Data Collection

Public announcements were made on Facebook, Instagram, and LINE groups. Eligibility was confirmed in telephone screening. After providing written informed consent, the participants completed a pretest survey, wherein their demographic characteristics were assessed and the POAS and CCPA scales were administered. The participants then completed the 60‐min SVVR intervention. After the intervention, the participants completed a posttest survey, wherein the POAS, CCPA scales, TAQ, and VRSQ were administered. Finally, semistructured interviews were conducted with each participant.

### 4.8 Data Analysis

Quantitative data were processed and analysed in SPSS (version 27; IBM, Armonk, NY, USA). Statistical significance was set at *p* < 0.05. Descriptive statistics—mean ± standard deviation and frequency (%) values—were used to summarise the data. The Kolmogorov–Smirnov test indicated that both POAS (*p* = 0.058) and CCPA (*p* = 0.200) scores followed a normal distribution; therefore, paired‐sample *t*‐tests were conducted to assess score changes.

Qualitative data were analysed using the six‐phase thematic analysis approach of Braun and Clarke (2006): familiarisation with the data, initial code generation, theme search, theme review, theme definition and naming, and report production. Coding and theme development were performed iteratively by two researchers to ensure dependability and credibility. Trustworthiness was further enhanced by the inclusion of direct quotations to ensure authenticity.

The quantitative and qualitative findings were integrated to identify convergence, divergence, and complementarity. The results were presented using joint displays (side‐by‐side tables and integrated result matrices) to highlight meta‐inferences and clarify how qualitative insights explained or expanded quantitative outcomes (Younas and Durante 2023).

### 4.9 Ethical Considerations

The present study adhered to the ethical principles outlined in the Declaration of Helsinki. This study protocol was approved by the Institutional Review Board of Tri‐Service General Hospital (reference number: A202405149; approval date: November 19, 2024). Written informed consent was obtained from all participants. All participants were informed of their right to withdraw from the study at any time. Participant confidentiality was maintained throughout data collection, and all data were anonymised before analysis. All digital data were encrypted, and interview recordings were anonymised before analysis.

## 5 Results

### 5.1 Participant Demographics

Table 1 presents the demographic characteristics of the study cohort. The participants' mean age was 35.13 ± 7.807 (range: 25–56) years. Most participants were women (*n* = 50 [92.6%]). Regarding workplace violence experience, 51.9% reported verbal abuse, 44.4% reported physical attacks, 31.5% reported threats, and 14.8% reported sexual harassment. Only 37.0% of the participants had previously received violence management training.

**TABLE 1:** Participant characteristics (*n* = 54).

Variable	*n*	%	Mean	Standard deviation	Range
Age (years)			35.13	7.807	25–56
Sex, *n* (%)					
Male	4	7.4			
Female	50	92.6			
Marital status, *n* (%)					
Single	32	59.3			
Married	22	40.7			
Education level, *n* (%)					
Junior college degree	10	18.5			
Bachelor of Science degree or higher	44	81.5			
Unit, *n* (%)					
Outpatient clinic	10	18.5			
General ward	9	16.7			
Psychiatric ward	7	13.0			
Emergency department	9	16.7			
Intensive care unit	6	11.1			
Operation room	8	14.8			
Haemodialysis unit	5	9.2			
Nursing ladder, *n* (%)					
N	4	7.4			
N1	16	29.6			
N2	19	35.2			
N3	8	14.8			
N4	7	13.0			
Nursing experience (years)			11.59	7.730	2–32
Workplace violence–related experience, *n* (%)					
Physical attacks	24	44.4			
Verbal abuse	28	51.9			
Threats	17	31.5			
Sexual harassment	8	14.8			
Previous violence management training, *n* (%)	20	37.0			

### 5.2 Intervention Feasibility

All participants completed the intervention, the pretest and posttest surveys, and the semistructured interviews. No participants withdrew from the study. A total of 15 participants (27.8%) obtained a VRSQ score of 0 out of 100. The remaining 39 participants had minimum, maximum, and average VRSQ scores of 4.17, 43.33, and 11.20, respectively. The average oculomotor subscale score was 15.1 (interquartile range: 0–25.0), and the average disorientation subscale score was 7.3 (interquartile range: 0–13.3; Table 2). No participants stopped using the SVVR system because of adverse physical symptoms.

**TABLE 2:** Total and subscale scores on Virtual Reality Sickness Questionnaire and Technology Acceptance Questionnaire (*n* = 54).

Variable	Mean score (standard deviation)	Median score	Interquartile range
Virtual reality sickness	11.20 (10.734)	9.58	0–17.08
Oculomotor subscale	15.12 (14.119)	8.33	0–25.00
Disorientation subscale	7.28 (10.219)	6.67	0–13.33
Technology acceptance	61.74 (4.919)	64.50	60.00–65.00
Perceived usefulness subscale	28.87 (2.056)	30.00	28.75–30.00
Perceived ease of use subscale	32.87 (3.802)	35.00	32.75–35.00

### 5.3 Quantitative Findings

The intervention significantly improved the participants' workplace violence–related perceptions and their confidence in managing incidents of violence (Table 3). POAS scores significantly increased from 67.37 ± 6.99 before the intervention to 75.76 ± 8.24 after the intervention (mean difference: 8.39; *t* = 9.62; *p* < 0.001). Similarly, CCPA scores significantly increased from 60.04 ± 8.46 before the intervention to 71.93 ± 7.84 after the intervention (mean difference: 11.89; *t* = 12.91; *p* < 0.001). The average TAQ score was high (61.74 ± 4.92). Specifically, the average scores on the perceived usefulness and perceived ease of use subscales were 28.87 ± 2.06 and 32.87 ± 3.80, respectively, indicating overall positive feedback on the usability and relevance of the SVVR system.

**TABLE 3:** Changes in attitudes towards and confidence in managing workplace violence (*n* = 54).

Outcome variable	Pretest score, mean (standard deviation)	Posttest score, mean (standard deviation)	*t*	*p*
Attitude	67.37 (6.989)	75.76 (8.244)	6.64	< 0.001
Confidence	60.04 (8.456)	71.93 (7.840)	12.91	< 0.001

### 5.4 Qualitative Findings

Analysis of interview recordings yielded 4 themes and 14 subthemes reflecting the participants' experiential learning.

#### 5.4.1 *Theme 1: Instructional* C*haracteristics of the Intervention*

##### 5.4.1.1 Immersive Presence

The participants reported that entering the 360° virtual scenario through a head‐mounted display created a strong sense of presence, making them feel as though they were physically present at the scene of the incident.

The experience was highly immersive and created a strong sense of presence. (N4)

I felt as if I were physically present, witnessing the incident unfold in real time. (N34)

The immersive nature of the intervention enabled me to actively engage with the scenario rather than merely absorb information. (N52)

##### 5.4.1.2 Emotional Resonance

The participants described forming an emotional connection with the characters depicted in the virtual scenario, experiencing the tension and psychological stress associated with the incident.

I could sense the oppressive atmosphere of medical violence as though I were physically present at the scene. (N28)

The experience fully immersed me in the scenario, enabling me to feel the immediate pressure and challenges. (N48)

I could truly feel the stress experienced by the onsite personnel. It felt incredibly real—almost as if I were the one managing the patient. (N53)

##### 5.4.1.3 Comprehensible Content

The participants indicated that observing how the nurse in the virtual scenario responded to the situation enhanced their understanding of the course concepts.

The video clearly demonstrated the nurse's real‐time actions and how the situation was handled. (N12)

This learning approach is simple and intuitive. It effectively conveys the content, unlike previous text‐heavy training. (N23)

Previously, I didn't know how to respond to incidents of violence. I sometimes used incorrect strategies. This session clarified appropriate responses—it was straightforward and easy to grasp. (N27)

#### 5.4.2 Theme 2: Advantages of the Intervention

##### 5.4.2.1 Enhanced Attention and Engagement

The participants consistently reported that the interactive and immersive features of the SVVR intervention facilitated sustained attention and enhanced learning effectiveness.

The script was vivid and well‐performed. The script helped me stay focused and increased both my motivation and interest. (N9)

The scenario was dynamic and interactive, fostering participation. I could stay focused and absorb the material quickly. (N17)

The interactive elements sustained my interest and helped me quickly grasp how to manage incidents of medical violence. (N48)

##### 5.4.2.2 Practical Experience

The participants reported feeling immersed in the management of a virtual incident of violence through the system's voice interactions and scenario‐based simulation.

It felt like I was really managing a violent patient again—carrying out full response procedures and learning preventive strategies. (N44)

In the virtual environment, I practiced different response strategies and gained relevant experience. (N25)

The training provided a realistic simulation and an opportunity to practice how to respond effectively. (N31)

##### 5.4.2.3 Self‐Reflection

While engaging with the virtual scenario, the participants reflected on their previous experiences managing violent patients.

I focused on the nurse's responses in the video. Her performance served as a good example and prompted me to reconsider how I respond. (N22)

The interactive questions deepened my thinking. I had to plan my response during the training. It wasn't just knowledge; it helped simulate real clinical responses. (N48)

The interactive question‐and‐answer format prompted me to think more critically and reflect on whether my typical responses were appropriate. (N11)

##### 5.4.2.4 Reinforced Memory Retention

The participants reported that the 360° panoramic video, voice interactions, and postintervention debriefing enhanced their ability to retain content related to violence management.

Having video references, interactive questions, and a summary made the content memorable. (N7)

This approach was more effective than just watching a video or lecture. It helped me recall response strategies and created mental imagery for future use. (N36)

This method made it easy to remember key techniques. I'll be able to respond more efficiently next time. (N51)

#### 5.4.3 Theme 3: Increased Competence in Violence Management

##### 5.4.3.1 Enhanced Risk Recognition

The participants indicated that the SVVR intervention enhanced their ability to recognise early indicators of patient aggression and implement preventive measures in advance.

I could observe the patient's behaviours and facial expressions in the video. This helped me spot warning signs and intervene early. (N32)

I learned how to detect subtle signs of aggression and proactively request assistance. (N50)

##### 5.4.3.2 Familiarity With Response Procedures

The participants indicated that the scenario enabled them to devise concrete strategies for managing incidents of violence.

I learned how to safely position myself, effectively communicate, and take protective measures. (N38)

I now understand each staff member's responsibilities during an incident of violence and how they typically respond. (N36)

##### 5.4.3.3 Enhanced Confidence

The participants stated that the SVVR intervention increased their confidence in both preventing and responding to incidents of violence.

Experiencing the scenario firsthand improved my memory and helped me respond faster in real life. (N28)

Retaining these methods has improved my response capabilities and increased my confidence. (N33)

When similar incidents occur, the scenario I practiced will come to mind and guide my response. (N37)

#### 5.4.4 Theme 4: Limitations of the Intervention

##### 5.4.4.1 Visual Discomfort

Some participants reported that the placement and font of the subtitles caused dizziness.

The subtitles were low and curved. If I needed to read them, it made me dizzy. (N5)

Sometimes the image was blurry and hard to focus on. (N10)

Sometimes I found it difficult to focus because the image appeared blurry. (N10)

My myopia may have worsened recently—some details weren't very clear. (N42)

##### 5.4.4.2 Inconsistent Speech Recognition

The voice sensor appeared somewhat unresponsive, occasionally generating inaccurate responses. Enhancing the speech recognition function could improve the smoothness of the learning experience. (N51)

##### 5.4.4.3 Headset Discomfort

The headset was not customised to individual face shapes, resulting in slight discomfort during use, but the discomfort was acceptable. (N44)

##### 5.4.4.4 Difficulty Operating System

Older individuals may require additional time and effort in learning how to operate the system, which may complicate the learning task. (N1)

### 5.5 Integration of the Findings

As shown in Table 4, the integrated quantitative and qualitative findings indicated that the SVVR intervention was both feasible and well accepted. The intervention effectively enhanced nurses' competence and confidence in managing workplace violence. The quantitative findings revealed significant improvements in POAS and CCPA scores, and the qualitative findings explained these improvements by clarifying that immersive realism, emotional engagement, and reflective opportunities provided by the 360° virtual environment increased situational awareness and reinforced skill acquisition. Minor divergences emerged regarding technical limitations, which contextualised the low VRSQ scores. Taken together, our results suggest that SVVR is a practical and pedagogically valuable tool that extends beyond conventional training by engaging nurses cognitively, emotionally, and behaviourally in violence management scenarios.

**TABLE 4:** Integrated quantitative and qualitative findings.

Domain	Quantitative findings	Qualitative findings	Mixed‐methods meta‐inferences
Feasibility	100% completion	Visual discomfort Inconsistent speech recognition Headset discomfort Difficulty operating system	Discordance: The quantitative findings confirm feasibility and tolerance (100% retention; minimal cybersickness), but the qualitative findings suggest that ergonomic and technical refinements are required for broader adoption
	Low Virtual Reality Sickness Questionnaire scores (mean score: 11.2/100)		
Effects	Significant postintervention improvements in the Perception of Aggression Scale score (+8.39; *p* < 0.001) and the Coping with Patient Aggression scale score (+11.89; *p* < 0.001)	Immersive presence Emotional resonance Enhanced risk recognition Familiarity with response procedures Increased confidence	Convergence: Quantitative gains in attitudes and confidence align with qualitative insights, indicating that immersive, practice‐based learning increases both situational awareness and self‐efficacy
Acceptance	High Technology Acceptance Questionnaire scores: perceived usefulness subscale (mean score: 28.87/30) and perceived ease of use subscale (mean score: 32.87/35)	Comprehensible content Enhanced attention and engagement Practical experience Self‐reflection Reinforced memory retention	Convergence: High quantitative acceptance corroborated by qualitative accounts of engagement, intuitive use, and memorable design, underscoring the pedagogical relevance of spherical video–based virtual reality in violence management training

## 6 Discussion

### 6.1 Principal Findings Versus the Literature

This convergent mixed‐methods study integrated quantitative and qualitative data to evaluate the feasibility, effectiveness, and acceptability of an SVVR workplace violence management training programme for nurses. The quantitative findings indicated high completion, strong acceptance, and significant gains in attitudes and confidence in managing workplace violence, and the qualitative results highlighted the mechanisms underlying these benefits—namely, immersive presence, emotional resonance, reflective learning, and experiential practice. This combination provided richer insights than either strand alone: quantitative scores unveiled measurable gains, and qualitative narratives explained why and how the intervention fostered skill acquisition, confidence, and motivation.

A notable area of convergence was observed between the improvements in POAS and CCPA scores and the qualitative findings of enhanced situational awareness, risk recognition, and self‐efficacy. Divergence occurred in the feasibility domain: although quantitative data indicated full completion and low VRSQ scores, qualitative data highlighted discomfort with subtitles, device fit, and inconsistent speech recognition. This discordance underscores the importance of combining methods, given that quantitative data alone may obscure practical refinements required for broader implementation.

All participants completed the SVVR intervention and the associated quantitative and qualitative assessments, indicating the strong feasibility and practicality of the programme design and research procedures. The high participation rate likely resulted from the alignment of the programme content with nurses' clinical responsibilities, the use of scenarios based on real cases, and the incorporation of innovative technology, which effectively enhanced interest and learning motivation (Zhang et al. 2021). Participants who reported mild dizziness when reading subtitles had low overall VRSQ scores. Biniok et al. (2024) reported a positive correlation between dizziness symptoms and age in a German cohort. Our participants were aged between 25 and 56 years. Discomfort perceived in this study might have been influenced by age and visual acuity. Future SVVR training programmes should continue to monitor and promptly address symptoms of dizziness to ensure optimal learning outcomes.

Our quantitative findings suggested that the SVVR intervention considerably enhanced the nurses' awareness of workplace violence and their confidence in handling such incidents. These results are consistent with those of a study suggesting that simulation‐based training enhances learning outcomes among nurses (Story et al. 2020). By combining 360° panoramic visuals with voice interactions, the SVVR intervention enabled learners to not only observe scenarios of violence but also actively engage in managing them, thereby deepening understanding and supporting the internalisation of effective response strategies. This finding aligns with that of a systematic review (Alsharari et al. 2025), which highlighted the role of virtual reality in fostering problem‐solving, communication, and professional skill development.

The qualitative data revealed that the SVVR intervention evoked strong emotional resonance and prompted self‐reflection, encouraging the participants to critically assess and adjust their response behaviours. This process aligns with Bandura's social cognitive theory, which posits that learning requires the sequential stages of attention, retention, reproduction, and motivation to translate knowledge into concrete action (Lavoie et al. 2018). Therefore, the course design effectively cultivated learners' meta‐cognitive processes and helped them develop competencies in managing high‐risk situations.

The TAQ scores indicated that the participants regarded the SVVR intervention as highly useful and user‐friendly. The intervention's flexibility, unrestricted by time or location, enabled the nurses to engage in repeated practice sessions, a learning modality consistent with adult learning theories that emphasize self‐directed learning. In addition, the incorporation of 360° visual perspectives and voice interactions promoted critical thinking and increased learners' confidence in making real‐time decisions, thereby enhancing motivation and acceptance (Baysan et al. 2023).

The participants highlighted several technical limitations, including subtitle placement, speech recognition accuracy, and device ergonomics; these concerns are consistent with those reported by Huang et al. (2022). Similarly, Lee et al. (2020) revealed that inadequate video quality and poorly optimised subtitles could detract from the overall learning experience in SVVR‐assisted education. Therefore, future programme design should focus on refining the user interface by improving subtitle placement, enhancing speech recognition functionality, and optimising device ergonomics to create a seamless and effective learning environment.

### 6.2 Strengths and Limitations

The strength of this study lies in its integrated mixed‐methods approach, which not only revealed improvements in the participants' workplace violence preparedness but also explained the underlying experiential and pedagogical processes. The results position SVVR as a highly feasible and innovative training modality that is particularly well suited to preparing nurses for high‐risk, low‐frequency, and high‐impact clinical situations, such as suicide prevention, child abuse reporting, and high‐stakes communication.

This study has some limitations. First, the use of a single‐group pretest–posttest design without a control group precluded causal inferences regarding the intervention's effectiveness. Second, the sample was drawn exclusively from a single medical centre, which restricts the generalisability of the findings. Finally, this study focused solely on short‐term learning responses and did not assess the sustainability of observed behavioural changes.

### 6.3 Recommendations for Future Research

In the future, multicentre randomised controlled trials should be conducted to generate causal inferences and to increase generalisability. Longitudinal designs are warranted to assess the retention and transferability of skills acquired through the SVVR intervention to clinical practice. In addition, further mixed‐methods research should be conducted to explore additional outcomes, such as cost‐effectiveness and scalability, and to refine technical aspects such as subtitle placement, device ergonomics, and speech recognition accuracy.

### 6.4 Implications for Policy and Practice

Our integrated findings highlight SVVR as a learner‐centred, immersive, and effective training modality for preparing nurses to manage workplace violence. For policies, the findings suggest that immersive training can be institutionalised as part of continual professional development and workforce resilience initiatives. For education, the added value lies in bridging theoretical knowledge with realistic practice, encouraging both cognitive and affective engagement. Extending SVVR modules to other high‐risk clinical contexts, such as suicide prevention, child maltreatment reporting, and disaster response, may further improve preparedness.

## 7 Conclusion

The present study suggests that SVVR‐assisted training is a feasible, acceptable, and effective approach for enhancing nurses' attitudes, confidence, and self‐efficacy in managing workplace violence. By integrating quantitative gains with qualitative insights into immersive learning processes, this study underscores SVVR's potential as an innovative alternative to conventional instruction. Integration of quantitative and qualitative data revealed both convergence (enhanced skills and confidence) and divergence (technical discomfort despite low VRSQ scores), offering a nuanced understanding of intervention feasibility. Overall, SVVR‐assisted training holds promise for advancing nurse education, policy, and workforce preparedness.

## Author Contributions

Study design: Y.‐Y.L., T.‐T.L., Y.‐L.H., H.‐P.F., C.‐Y.L., W.‐C.T. Data collection: Y.‐Y.L., Y.‐L.H., W.‐C.T. Data analysis: Y.‐Y.L., T.‐T.L., H.‐P.F., W.‐C.T. Study supervision: C.‐Y.L., W.‐C.T. Manuscript writing: Y.‐Y.L., T.‐T.L., Y.‐L.H., H.‐P.F., C.‐Y.L., W.‐C.T. Critical revisions for important intellectual content: W.‐C.T. All listed authors approved the final version for submission.

## Funding

This study was supported by the National Science and Technology Council (grant number: NSTC 113‐2314‐B‐016‐019‐MY3). The funder was not involved in the study design, data collection, data analysis and interpretation, writing of the manuscript, or decision to submit this article for publication.

## Ethics Statement

This study was not a clinical trial and therefore was not prospectively registered. Ethical approval was obtained from the institutional review board of the study hospital before data collection. All study procedures complied with relevant ethical guidelines.

## Conflicts of Interest

The authors declare no conflicts of interest.

## Data Availability

The data that support the findings of this study are available on request from the corresponding author. The data are not publicly available due to privacy or ethical restrictions.
